# Positive effects of Mulberry leaf extract on egg quality, lipid metabolism, serum biochemistry, and antioxidant indices of laying hens

**DOI:** 10.3389/fvets.2022.1005643

**Published:** 2022-09-16

**Authors:** Bo Zhang, Zeben Wang, Chenxuan Huang, Dehe Wang, Dongmei Chang, Xiaowei Shi, Yifan Chen, Hui Chen

**Affiliations:** ^1^College of Animal Science and Technology, Hebei Agricultural University, Baoding, China; ^2^Agricultural and Animal Husbandry Technology Extension Station in Tong Town, Shaanxi Province, Yulin, China; ^3^College of Management Science and Engineering, Hebei University of Economics and Business, Shijiazhuang, China; ^4^Zhengding County Mulberry Industry Application Research Institute, Shijiazhuang, China

**Keywords:** mulberry leaf extract (MLE), laying hen, egg quality, antioxidant indexes, lipid metabolism

## Abstract

Plant extracts are becoming a hot topic of research by animal husbandry practitioners following the implementation of a global policy to restrict antibiotic use in animal production. Mulberry leaf extract has received considerable attention as a new plant extract. Mulberry leaf polysaccharides and flavonoids are its main constituents, and these substances possess immunoregulatory, hypoglycemic, antioxidant, and anticoagulant properties. It is however less common to use them in poultry production. Therefore, we investigated the effects of adding MLE to the diet of laying hens on egg quality, lipid metabolism, serum biochemistry, and antioxidant indices in this study. A total of 288 Lohmann Silber layers, aged 38 weeks, were randomly assigned to four groups (six replicates of 12 hens each). Hens were fed a basal diet supplemented with 0 (control diet), 0.4, 0.8, or 1.2% MLE for 56 d. Results showed that the addition of 0.4–1.2% MLE to the diet improved aspartate transaminase (AST) activity in the serum of laying hens, reduced low-density lipoprotein (LDL-C) content in the serum, and significantly decreased yolk triglyceride (TG) and total cholesterol (TC) contents (*P* < 0.05). No adverse effects were observed on production performance (*P* > 0.10). MLE (0.4 and 1.2%) significantly reduced the TG and TC levels in the liver (*P* < 0.05). MLE (0.8 and 1.2%) significantly increased glutathione peroxidase (GSH-Px) activity in the serum, decreased alanine transaminase (ALT) activity, TG and TC content in the serum, and improved egg yolk color (*P* < 0.05). MLE (1.2%) significantly increased high-density lipoprotein (HDL-C) content and superoxide dismutase (SOD) activity in the serum and enhanced eggshell strength (*P* < 0.05). The liver-related lipid metabolism gene assay revealed that the relative mRNA expression of PPARα and SIRT1 in the liver was significantly upregulated and that of FASN and PPARγ was significantly decreased after the addition of MLE. In contrast, the relative mRNA expression of SREBP-1c in the liver dramatically decreased after the addition of 0.8 and 1.2% MLE (*P* < 0.05). The addition of MLE to the diet improved egg quality and the economic value of hens by increasing antioxidant capacity and lipid metabolism. The most appropriate amount of MLE to be added to the diet of laying hens was 0.8%. Our study provides a theoretical reference for the application of MLE in egg production and to promote the healthy and sustainable development of the livestock and poultry industry under the background of antibiotic prohibition.

## Introduction

Modern egg farming has benefited from highly intensive farming methods, which increasing efficiency, convenience, and effectiveness for farmers. However, this has also put egg-laying hens at risk of inherited diseases related to lipid metabolism. The use of antibiotics to treat these diseases is not the best solution, and the concept of healthy consumption drives consumers to prefer purchasing green and antibiotic-free poultry products. Eggs are one of the most readily available high-quality proteins, but the lipid and high cholesterol content (~30% of the nutrient content) of egg yolks have become an issue of concern for consumers (1). Excessive cholesterol intake has adverse effects on the body and increases the risk of developing diabetes (2), especially for people with underlying diseases, such as heart disease (3). Therefore, finding alternatives to antibiotics to balance the product market demand has become a part of the modern farming industry.

Plant extracts have become a hot topic for industry research as a natural feed additive due to decrees issued by countries including China to restrict the growth of antibiotics in livestock production (4, 5). Most plants contain anti-nutritional elements, such as tannins and phytic acids, and chemical extraction can be used to eliminate these effects on livestock and poultry and improve the palatability of feed. Previous studies have shown that the addition of natural mineral elements, such as iodine and iron, to the diet of laying hens can improve egg quality (6, 7). The use of natural plant extracts for animal production has many benefits. The addition of 1.5 g/kg of ginger powder to the diet of Japanese quails improved their performance and egg quality (8). The addition of cinnamon oil to the diet of poultry can balance the gastrointestinal microenvironment, optimize lipid metabolism, and thus increase production performance and immune function (9). The addition of tartary buckwheat extract to the diet of ewes and lambs can alleviate oxidative stress and enhance production performance (10).

Mulberry (*Morus alba* L.) is a deciduous tree belonging to the family Moraceae. It is distributed worldwide, mostly in Asian countries, including China, Japan, and Korea, where is used in traditional industries, such as sericulture (11, 12). The leaves of mulberry plants contain biologically active substances, such as polysaccharides, flavonoids, and alkaloids, which contribute to lowering triglycerides, antioxidants, immunity, and so on (13). Mulberry leaf extract (MLE) has several applications in animal production. MLE can reduce blood glucose levels in mice, which is likely due to active ingredients that stimulate adipocyte proliferation and differentiation. Adipogenic transcription factors and downstream gene expression are likely regulated in the same manner (14). The addition of 200–1,600 mg/kg of mulberry leaf flavonoids to the diet of fattening pigs significantly improves their growth performance and meat quality and positively affects lipid metabolism (15). However, the application of MLE in egg production has not been extensively studied. The addition of 0.5% mulberry leaf powder to the diet of Hendrix hens can improve egg yolk weight, shell weight, Haugh unit, yolk color, and antioxidant status (16). It was found that 4 mg of mulberry leaf polysaccharide supplement fed to chicks vaccinated against Newcastle disease virus triggered an immune response and resulted in high levels of antibodies for several weeks post vaccination (17). The addition of 60 mg/kg mulberry leaf flavonoids to the diet of older breeders improved eggshell thickness and shell strength by affecting calcium transport in the shell glands (18). As of now, mulberry leaf extract is primarily used *in vitro* or on rats, with relatively few animal production studies and even fewer studies on laying hens. Therefore, this study aimed to investigate the effects of MLE on egg quality, antioxidants, and lipid metabolism. It also aimed to determine the optimal ratio of MLE in hen diets to provide a theoretical reference for the application of MLE in egg production and to promote the healthy and sustainable development of the livestock and poultry industry under the background of antibiotic prohibition.

## Materials and methods

### Birds, diets, and management

A single-factor design was used for the experiment. A total of 288 38-week-old Lohmann Silber layers with good health and similar growth were randomly divided into four groups, each dietary treatment had 6 replicates with 12 hens each. The pre-trial and trial periods were 14 and 56 d, respectively. All chickens were fed a basic diet during the pre-trial period. In the trial period, the control group was fed a basic diet, and the experimental group was fed a basic diet supplemented with various concentrations of MLE, namely 0.4, 0.8, and 1.2%. MLE was purchased as a dark green powder with silica as the carrier from Xiangda Hezhong Biotechnology Co., Ltd. (Hebei, China). The extraction method used was hot-water extraction, the main components were mulberry leaf polysaccharides (20%), mulberry leaf flavonoids (3%), and alkaloids (2%). The basal diet was formulated according to the NRC (1994) to meet the nutrient requirements of laying hens (19) (Table 1). The experimental site was the animal husbandry teaching base of Hebei Agricultural University, and laying hens were caged with three and a half open steps. Hens were allowed to eat and drink freely. Natural ventilation and natural and artificial light were used. The light/dark schedule was 16/8 h, and the light intensity used was 15 lx. Eggs were collected at 15:00 every day, and the mental state and death of the chickens were recorded. The chicken coop was cleaned regularly.

**Table 1:** Ingredients and chemical composition of basal diet.

**Items**	**Content/%**
**Ingredients**
Corn	66.40
Soybean meal	25.00
Wheat bran	2.20
Vegetable oil	0.80
NaCl	0.30
CaHPO_4_	1.50
Fish meal	2.80
Premix^a^	1.00
Total	100.00
**Nutrient levels** ^**b**^
ME/(MJ/kg)	12.38
CP	16.57
Ca	3.60
AP	0.45
Lys	0.86
Met	0.38

^a^The premix provided the following per kg of the diet: VA 12 000 IU, VB_1_ 6 mg, VB_2_ 7 mg, VB_6_ 7 mg, VB_12_ 0.34 mg, VD 4 500 IU, VE 20 IU, VK 3.2 mg, biotin 5 mg, folic acid 1.1 mg, nicotinic acid 50 mg, Cu (as copper sulfate) 9 mg, Fe (as ferrous sulfate) 50 mg, Mn (as manganese sulfate) 100 mg, Zn (as zinc sulfate) 85 mg, I (as potassium iodide) 90 mg, Se (as sodium selenite) 0.30 mg.

^b^Nutrient levels were all calculated values.

### Sample collection

Two chickens were randomly selected from each replicate on the 56th day of the experiment, and 48 chickens were fasted for 24 h with free access to water. Afterwards, blood was collected from the wing vein, kept at 20–25°C, centrifuged at 3,000 rpm for 15 min, and the supernatant was stored at −20°C. These chickens were then slaughtered according to the animal welfare slaughtering procedure, the livers were removed and weighed, and ~2 g of the left side of the livers were snap frozen in liquid nitrogen. The frozen liver samples were stored at −80°C.

### Egg quality characteristics

Twenty eggs were randomly selected from each group on day 28 and 58 of the experiment to determine the egg quality. Eggshell strength was measured using an egg force reader (EFR-01, ORKA Technology Co., Ltd., Herzliya, Israel); egg yolk color was measured using a yolk color chart (Robotmation, Co., Ltd., Tokyo, Japan); Vernier calipers were used to measure eggshell thickness at the blunt end, sharp end, and middle part after the eggshell membrane was peeled off, and the average thickness value was determined. Egg long and short diameters were measured using an egg form coefficient measuring instrument (NFN385, FHK Corp., Tokyo, Japan), the ratio of long diameter to short diameter was measured using an egg shape index, and protein height and Haugh unit was measured using an egg multitester (model EA-01, ORKA Technology Co., Ltd., Herzliya, Israel) (20). The yolk was separated and weighed, the proportion of yolk was calculated, and the yolk moisture content was calculated by mixing three yolks and then freeze-drying (21).

### Plasma indices

An enzyme labeling instrument (Bio Tek Instruments, Inc., Vermont, VT, USA) was used to determine the levels of serum albumin (ALB; cat. NO. A045-3-2), malondialdehyde (MDA; Cat. NO. A003-1-2), total protein (TP; Cat. NO. A045-3-2), triglyceride (TG; cat. NO. A110-1-1), total cholesterol (TC; Cat. NO. A111-1-1), high-density lipoprotein cholesterol (HDL-C; Cat. NO. A112-1-1), low-density lipoprotein cholesterol (LDL-C; Cat. NO. A113-1-1), very-low-density lipoprotein (VLDL; cat. NO. JL15942), aspartate transaminase (AST; cat. NO.C010-2-1), alanine transaminase (ALT; cat. NO.C09-2-1), superoxide dismutase (SOD; Cat. NO. A001-3-2), glutathione peroxidase (GSH-Px; cat. NO. A005-1-2), catalase activity (CAT; Cat. NO. A007-1-1), and total antioxidant capacity (T-AOC; cat. NO. A015-1-2). VLDL kits were purchased from Shanghai Jianglai Biotechnology Co. Ltd. (Shanghai, China). The remaining kits were purchased from Nanjing Jiancheng Bioengineering Institute (Nanjing, Jiangsu, P.R. China). These kits were used according to the manufacturer's instructions (22, 23).

### Liver and egg yolk lipid analysis

The liver samples were thawed, homogenized at a constant temperature of 0°C using a high-speed homogenizer, and the TG and TC levels were determined. The freeze-dried egg yolks were homogenized at a constant temperature of 0°C, and the TG and TC levels were determined (24).

### Gene expression

Quantitative real-time PCR was performed to analyze the relative mRNA expression of genes related to liver lipid metabolism. Primers used in this study are listed in Table 2. The β-actin gene was used as an internal reference. Real-time PCR was performed using a fluorescence quantitative PCR system (SLAN-96P, Shanghai Hongshi Medical Technology Co., Ltd., Shanghai, China). Relative mRNA expression of related genes was analyzed using the 2^−ΔΔCt^ method (16). Quantitative real-time PCR was performed by the Huaying Institute of Biotechnology in Beijing, China (25).

**Table 2:** List of gene primer sequences.

**Genes**	**NCBI ID no**.	**Primer sequence (5^′^-3^′^)**	**Product length (bp)**
*PPARα*	NM_001001464.1	F-AGTAAGCTCTCAGAAACTTTGTTG	108
		R-ACATTGGTGATAGCAAGTGGC	
*PPARγ*	NM_001001460.1	F-CCAGCGACATCGACCAGTTA	109
		R-CTTGCCTTGGCTTTGGTCAG	
*FASN*	NM_205155.4	F-GCGGGCAAAGACTCACAATG	112
		R-GGTGCGGTGATCTCCTTCAA	
*SIRT1*	NM_001004767.2	F-CTTCTCCAAGATGGCGGACG	120
		R-CCGTCTTCCGAGTTCAGGC	
*SREBP-1C (SREBF1)*	NM_204126.3	F-GAGCACCTCCTGGAGAAAGC	88
		R-CATCCGAAAAGCACCCCTCT	
*β-actin*	NM_205518.2	F-CGGACTGTTACCAACACCCA	115
		R-TCCTGAGTCAAGCGCCAAAA	

PPARα, peroxisome proliferators-activated receptor-α; PPARγ, peroxisome proliferators-activated receptor-γ; SIRT1, Silent information regulator 1; FASN, fatty acid synthetase; SREBP-1c(SREBF1), sterol regulatory element-binding protein-1c.

### Statistical analysis

The data were analyzed using one-way ANOVA (LSD) with Duncan's method for multiple comparisons between groups (26). Orthogonal polynomial contrasts were used to estimate the linear and quadratic effects of the various amounts of MLE added. All data were analyzed using SPSS (version 25.0; IBM Inc., New York, US), and images were created using GraphPad Prism version 8.0.2 for Windows (GraphPad Software, La Jolla California USA, www.graphpad.com). The results are presented as the mean ± standard deviation (*SD*), and statistical significance was set at *P* < 0.05 (27).

## Results

### Serum biochemical indices

The addition of MLE to the diet significantly reduced serum AST activity and LDL-C levels (*P* < 0.05) (Table 3). The addition of 0.8 and 1.2% MLE to the diet significantly increased serum ALT activity (*P* = 0.006) and decreased the TG (*P* < 0.05) and TC content (*P* < 0.05), whereas the addition of 1.2% MLE significantly increased serum HDL-C content (*P* < 0.05). The serum levels of TG, TC, AST, and ALT decreased significantly, and HDL-C increased significantly (linear or quadratic, *P* < 0.05) with increasing levels of Mulberry leaf extract in the diet. No statistically significant differences were found in other serum indicators (*P* > 0.10).

**Table 3:** Effects of mulberry leaf extract on serum biochemical indices of laying hens.

**Items**	**Control group**	**Mulberry leaf extract added levels (%)**			***P*** **-value**		
		**0.4**	**0.8**	**1.2**	**ANOVA**	**Linear**	**Quadratic**
ALB/(g/L)	26.14 ± 2.25	26.33 ± 1.47	26.89 ± 2.06	28.11 ± 2.19	0.224	0.046	0.108
GLB/(μg/mL)	49.05 ± 5.45	49.50 ± 4.19	50.36 ± 2.79	51.83 ± 2.41	0.512	0.134	0.310
TG/(mmol/L)	23.27 ± 2.08^a^	22.99 ± 2.12^a^	21.58 ± 1.95^ab^	20.54 ± 2.01^ab^	0.041	0.005	0.017
TC/(mmol/L)	4.55 ± 0.65^a^	4.36 ± 0.51^ab^	4.01 ± 0.39^bc^	3.75 ± 0.32^c^	0.013	0.001	0.004
VLDL-C/(mmol/L)	8.42 ± 2.07	7.44 ± 2.61	7.36 ± 1.26	6.80 ± 1.37	0.442	0.541	0.092
LDL-C/(mmol/L)	0.64 ± 0.05^a^	0.51 ± 0.06^c^	0.59 ± 0.06^b^	0.58 ± 0.05^b^	<0.001	0.277	0.007
HDL-C/(mmol/L)	0.85 ± 0.12^b^	0.86 ± 0.03^b^	0.89 ± 0.08^b^	0.97 ± 0.11^a^	<0.001	<0.001	<0.001
ALT/(U/L)	310.58 ± 25.32^a^	296.24 ± 28.16^ab^	279.25 ± 25.83^bc^	263.25 ± 24.02^c^	0.006	<0.001	0.002
AST/(U/L)	64.85 ± 7.21^a^	58.41 ± 6.13^b^	56.97 ± 5.13^b^	53.15 ± 4.13^b^	0.004	<0.001	0.001

Different letter in each row indicates a significant difference (p < 0.05).

ALB, albumin; GLB, globulin; TG, triglyceride; TC, total cholesterol; VLDL-C, very-low-density lipoprotein; LDL-C, Low-Density Lipoprotein; HDL-C, high-density lipoprotein; ALT, alanine transaminase; AST, aspartate transaminase.

### Egg quality

The egg quality-related characteristics are listed in Table 4. Up to day 28, the addition of 1.2% MLE to the diet significantly improved the eggshell strength (*P* < 0.05), and the addition of 0.8 and 1.2% MLE significantly improved the egg yolk color (*P* < 0.05). The yolk color and protein height increased significantly, and yolk weight decreased significantly (linear *P* < 0.05) as the level of MLE added to the diet increased. However, other characteristics were not statistically different (*P* > 0.10). Up to day 56, the addition of 1.2% MLE to the diet significantly improved eggshell strength (*P* < 0.05) and egg yolk color (*P* < 0.05). The yolk percentage decreased significantly (linear *P* < 0.05), and the yolk color increased significantly (quadratic, *P* < 0.05) with increasing levels of MLE added to the diets.

**Table 4:** Effects of mulberry leaf extract on egg quality of laying hens.

**Items**	**Control group**	**Mulberry leaf extract added levels (%)**			***P***		
		**0.4**	**0.8**	**1.2**	**ANOVA**	**Linear**	**Quadratic**
**D28**
Egg weight/g	59.81 ± 4.11	59.35 ± 3.97	59.19 ± 4.30	59.85 ± 3.84	0.942	0.715	0.838
Eggshell strength/N	39.71 ± 8.43^b^	41.08 ± 5.81^b^	44.65 ± 7.93^ab^	47.20 ± 5.98^a^	0.010	0.082	0.186
Yolk color	8.33 ± 0.91^b^	8.72 ± 0.67^ab^	8.94 ± 0.80^a^	9.00 ± 0.69^a^	0.049	0.023	0.053
Eggshell thickness/mm	0.34 ± 0.26	0.35 ± 0.26	0.36 ± 0.18	0.35 ± 0.15	0.109	0.232	0.141
Egg shape index	1.32 ± 0.04	1.33 ± 0.06	1.31 ± 0.03	1.32 ± 0.03	0.375	0.459	0.468
Haugh unit	83.48 ± 5.49	83.06 ± 5.47	83.31 ± 5.89	83.35 ± 6.14	0.997	0.633	0.626
Egg yolk weight	16.94 ± 1.72	16.65 ± 1.60	16.12 ± 1.00	16.03 ± 1.17	0.185	0.046	0.128
Eggshell weight	8.06 ± 0.84	7.90 ± 0.82	7.91 ± 0.51	8.33 ± 0.52	0.230	0.545	0.402
Protein height	6.00 ± 1.37	6.25 ± 1.33	6.67 ± 1.08	7.00 ± 1.17	0.205	0.049	0.122
Egg yolk ratio/(%)	27.78 ± 0.84	26.28 ± 0.90	27.18 ± 0.95	29.08 ± 3.81	0.153	0.110	0.127
Egg yolk moisture content/(%)	48.28 ± 5.12	48.51 ± 3.29	46.26 ± 4.70	44.13 ± 4.51	0.313	0.738	0.217
**D56**
Egg weight/g	59.46 ± 4.44	59.15 ± 4.15	59.73 ± 4.94	59.49 ± 4.12	0.985	0.930	0.924
Eggshell strength/N	39.73 ± 4.47^b^	42.64 ± 5.30^ab^	43.25 ± 5.68^ab^	44.58 ± 5.35^a^	0.048	0.233	0.040
Yolk color	9.56 ± 0.78^b^	10.17 ± 0.98^a^	10.33 ± 1.03^a^	10.50 ± 0.62^a^	0.006	0.125	0.011
Eggshell thickness/mm	0.34 ± 0.03	0.36 ± 0.03	0.36 ± 0.02	0.34 ± 0.03	0.219	0.076	0.206
Egg shape index	1.32 ± 0.23	1.33 ± 0.23	1.29 ± 0.21	1.31 ± 0.15	0.931	0.968	0.825
Haugh unit	77.10 ± 6.54	77.85 ± 7.23	78.17 ± 7.97	77.99 ± 6.11	0.131	0.393	0.424
Egg yolk weight	18.63 ± 2.41	18.32 ± 1.71	18.53 ± 1.45	18.73 ± 1.48	0.891	0.360	0.632
Eggshell weight	7.78 ± 0.70	7.86 ± 0.63	7.99 ± 0.45	7.85 ± 0.65	0.791	0.360	0.411
Protein height	5.90 ± 1.62	5.76 ± 2.09	6.05 ± 1.50	6.20 ± 1.38	0.869	0.642	0.774
Egg yolk ratio/(%)	27.85 ± 1.03	26.51 ± 0.88	27.16 ± 0.91	27.33 ± 0.71	0.110	0.014	0.052
Egg yolk moisture content/(%)	48.32 ± 3.72	48.41 ± 2.81	46.50 ± 5.88	43.89 ± 9.13	0.523	0.793	0.058

Different letter in each row indicates a significant difference (p < 0.05).

### Serum antioxidant capacity

The indicators related to the serum antioxidant capacity are listed in Table 5. The addition of MLE to the diet, compared to the control group, tended to increase CAT activity (*P* = 0.057). The addition of 1.2% MLE to the diet significantly increased SOD activity (*P* < 0.05), and the addition of 0.8 and 1.2% MLE to the diet significantly increased GSH-Px activity (*P* < 0.05). As the level of MLE added to the diet increased, serum CAT, SOD, and GSH-Px activities increased significantly (linear or quadratic, *P* < 0.05), T-AOC capacity increased significantly (linear *P* < 0.05), and MDA content increased significantly (quadratic, *P* < 0.05).

**Table 5:** Effects of mulberry leaf extract on serum antioxidant capacity of laying hens.

**Items**	**Control group**	**Mulberry leaf extract added levels (%)**			***P***		
		**0.4**	**0.8**	**1.2**	**ANOVA**	**Linear**	**Quadratic**
CAT/(U/mL)	18.25 ± 0.98	19.07 ± 1.51	19.87 ± 1.35	20.10 ± 1.71	0.057	0.007	0.023
T-AOC/mM	3.54 ± 0.42	3.68 ± 0.41	4.02 ± 0.39	3.75 ± 0.42	0.121	0.018	0.057
SOD/(U/mL)	151.42 ± 8.96^b^	155.33 ± 10.77^b^	161.10 ± 7.29^ab^	165.64 ± 10.53^a^	0.029	0.002	0.010
GSH-Px/(U/mL)	108.36 ± 9.81^c^	115.74 ± 11.19^bc^	126.25 ± 13.32^ab^	130.25 ± 12.37^a^	0.003	<0.001	0.001
MDA/(nmol/mL)	8.82 ± 1.70	8.10 ± 1.13	7.37 ± 1.10	7.11 ± 1.83	0.151	0.492	0.006

Different letter in each row indicates a significant difference (p < 0.05).

CAT, catalase activity; T-AOC, total antioxidant capacity; SOD, superoxide dismutase; GSH-Px, glutathione peroxidase MDA, malondialdehyde.

### Liver and yolk lipid profile

Compared to the control group, the addition of MLE to the diet significantly reduced the TG (*P* < 0.05) and TC content in egg yolk (*P* < 0.05) (Table 6). The addition of 0.4 and 1.2% MLE to the diet significantly reduced the TG content in the liver (*P* < 0.05), and the addition of 0.4 and 0.8% MLE significantly reduced the TC content in the liver (*P* < 0.05). The TG content in the liver and the TG and TC contents in egg yolk showed linear and quadratic changes, respectively, with an increase of dietary MLE (*P* < 0.05).

**Table 6:** Effects of mulberry leaf extract on lipid metabolism parameters of laying hens.

**Location**	**Items**	**Control group**	**Mulberry leaf extract added levels (%)**			***P***		
			**0.4**	**0.8**	**1.2**	**ANOVA**	**Linear**	**Quadratic**
Liver	TG/(mmol/L)	2.68 ± 0.69^a^	1.76 ± 0.20^b^	2.06 ± 0.71^ab^	1.97 ± 0.65^b^	0.046	0.015	0.049
	TC/(mmol/L)	1.05 ± 0.19^a^	0.80 ± 0.08^b^	0.96 ± 0.17^b^	0.85 ± 0.20^ab^	0.052	0.062	0.177
Yolk	TG/(mmol/L)	4.07 ± 0.80^a^	3.02 ± 0.21^b^	2.56 ± 0.40^b^	2.92 ± 0.74^b^	0.002	0.014	0.001
	TC/(mmol/L)	5.31 ± 0.71^a^	1.54 ± 0.29^c^	3.71 ± 1.29^b^	3.95 ± 0.66^b^	<0.001	<0.001	<0.001

Different letter in each row indicates a significant difference (p < 0.05).

TG, triglyceride; TC, total cholesterol.

### Lipid metabolism

**Figure 1:**
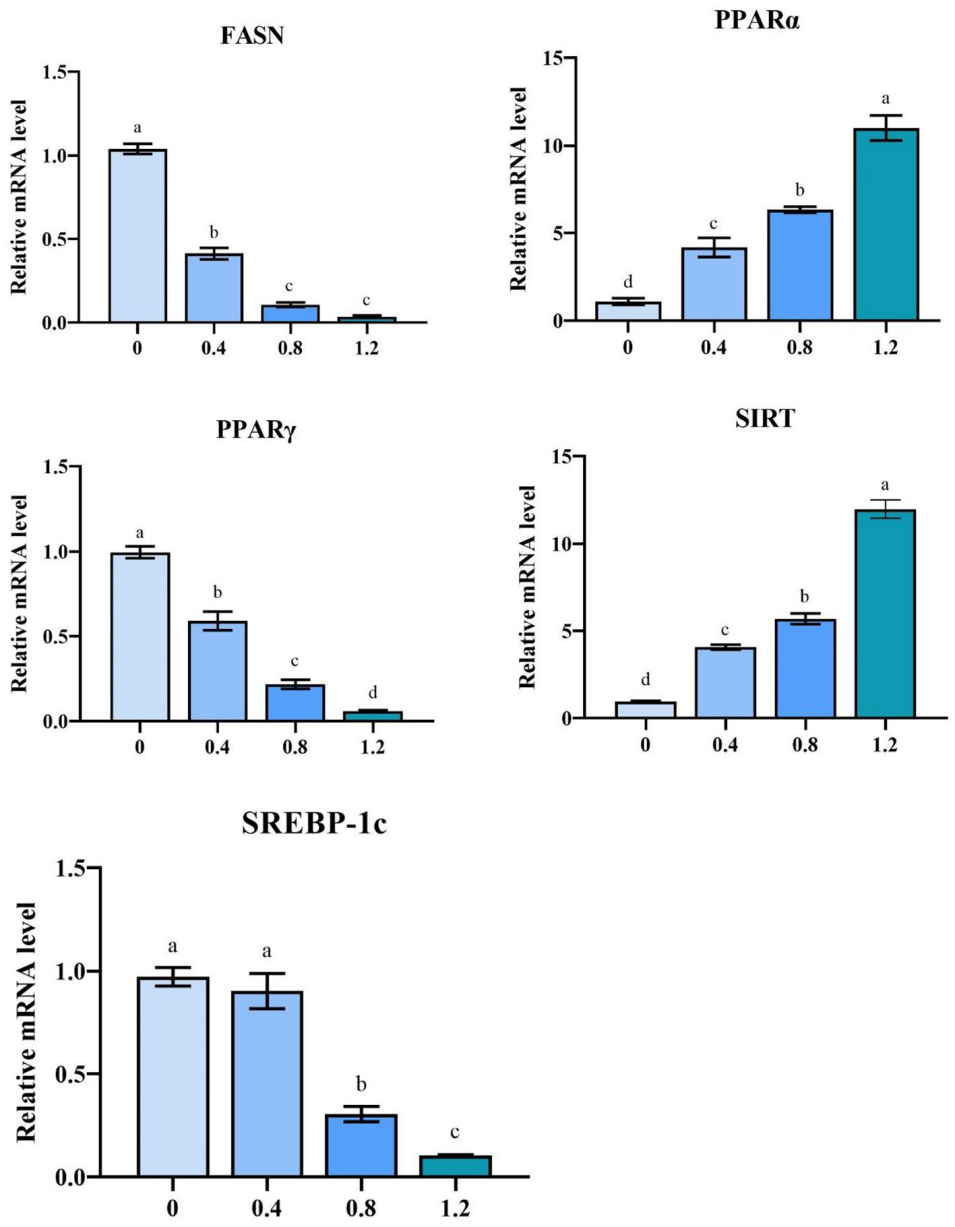
The effect of MLE on the mRNA expression of the laying hen hepatic (FASN, SIRT, PPARγ, SREBP-1c and PPARα) genes (mean ± MSE). Columns with different superscript letters are significantly different (*P* < 0.05).

The relative mRNA expression of PPARα and SIRT1 was significantly upregulated in MLE treatment groups than that in the control group (*P* < 0.05) (Figure 1). The relative mRNA expressions of FASN, PPARγ, and SREBP-1c were significantly decreased (*P* < 0.05) in the liver after the addition of MLE at 0.8 and 1.2%.

## Discussion

Mulberry trees are suitable for cultivation in most regions and have many uses, including for consumption, and ornate and medicinal uses. Biologically active substances such as polysaccharides, flavonoids, and alkaloids are extracted from mulberry leaves and can be used in several applications. Mulberry leaf polysaccharides have been shown to display a variety of pharmacological effects including antioxidant, hypoglycemic, and immune-boosting properties (17, 28). This study was conducted to evaluate the effects of MLE on laying hens with regards to serum biochemical parameters, egg quality, antioxidant properties, and lipid metabolism.

Serum biochemical indicators can reveal the metabolism and health status of body (29). ALT and AST activities are often used to determine the health status of the heart and liver. The transaminase activity of the liver is higher than that of the blood and the liver cell membrane ruptures during liver injury. The release of transaminase into the blood increases transaminase activity in the blood (30). Researchers have found that the addition of 1.0% Chinese herbal mixture to the diet of laying hens can reduce serum ALT contents (31). Salvia polysaccharides were added to drinking water at a concentration of 0.5–2.0 g/L and showed a significant reduction in ALT and AST activities in chicken serum (32). Our findings were consistent with the results of these studies. The AST and ALT activities decreased in the current study with MLE addition. These results combined with other data from this experiment show that mulberry leaf polysaccharides may exhibit antioxidative properties, which helps to reduce the liver damage caused by hens laying egg over a long period of time. Serum contents of TG, TC, LDL-C, and HDL-C were used to determine whether lipid metabolism in the animals was normal. High-energy diets fed to hens during the peak laying period can easily lead to lipid metabolism-related diseases in the late laying period, thereby reducing economic efficiency. A previous study showed that the addition of 0.5% MLE to the diet reduced serum TG, TC, and LDL-C levels in rats (33). The results from another study supported this finding (34). The present study demonstrated that the addition of MLE to the diet was associated with significant reductions in serum TG, TC, and LDL-C contents and significant increases in HDL-C contents. This indicates that the increase of lipolytic capacity may be due to the hypoglycemic effect of mulberry leaf polysaccharides. Studies suggested that mulberry leaf polysaccharides may reduce blood glucose by affecting the activity of related enzymes, improving glucose and lipid metabolism, and regulating the related lipid metabolism signaling pathways (35, 36). The cause of these results were further investigated.

Egg-laying hens exhibit fast metabolisms during the peak egg-laying period, which results in the rapid accumulation of a large number of free radicals in the body, leading to lipid peroxidation. This inhibits the activity of various antioxidant enzymes, causing oxidative stress and cellular tissue damage, resulting in accelerated aging of the body and adverse effects on production performance. Therefore, during peak egg production, we should take the initiative to alter the diet to avoid the premature aging of laying hens. The antioxidant enzymes SOD, GSH-Px, and CAT are the important parts of the *in vivo* antioxidant system. The T-AOC contents indicate the status of the non-enzymatic reactive oxygen defense system of body, whereas MDA contents reflect the rate and intensity of lipid peroxidation in the body (37). The addition of antioxidative substances to the feed will help improve the ability to scavenge free radicals of body and maintain the redox balance (38). According to the results of the experiment, mulberry leaf powder significantly increased the GSH-Px activity in the serum of Xiangcun black pigs (39). In the current study, supplementing the diet with MLE significantly increased the serum levels of SOD and GSH-Px, indicating that MLE has antioxidative properties. Many *in vitro* tests have demonstrated the scavenging effect of mulberry leaf polysaccharides on free radicals, such as 1,1-diphenyl-2-picrylhydrazyls (DPPH), hydroxyl (OH^−^), and superoxide (${\text{O}}_{2}^{-}$) (36, 40). It has been hypothesized that mulberry leaf polysaccharides also play an antioxidative role in laying hens.

In the production of modern laying hens, producers prefer using natural plant additives to obtain higher egg quality to comply with local regulations and policies on the use of additives. However, producers can improve egg quality and functional differences using other competing products to obtain great economic benefits. Studies have shown that dietary administration of 100 mg/kg of Yucca schidigera extract could significantly improve egg quality (41). Additionally, researchers have found that the addition of mulberry leaf powder to the diet significantly improved egg yolk color, but adding more than 10% mulberry leaf powder negatively affected egg quality (42). One study found that yolk weight, eggshell weight, eggshell strength, eggshell thickness, yolk color, and Haugh units increased in all MLE supplemented groups after adding 1% MLE to the diet of laying hens (16), which is consistent with the results of our present experiment, where adding MLE the diet caused egg quality-related indicators to be affected linearly and quadratically. The addition of mulberry leaf flavonoids to the diet enhanced eggshell strength by increasing the antioxidant capacity of the uterine shell gland and calcium deposition (significantly upregulating the expression of related genes, namely ESRpha, ESRbeta, KCNA1, OPN, CABP-28K, and CDH6) (18). In this current study, adding MLE to the diet improved shell strength and yolk color on days 28 and 56. Increasing eggshell strength within a certain range is beneficial for reducing the damage rate of eggs during transportation and reducing loss. The measures to improve the eggshell strength have been reported. This result may be related to the active ingredients, mulberry leaf polysaccharides, and mulberry leaf flavonoids in the MLE. The further research and confirmation were required. Yolk color is one of the most critical indicators of egg quality. There is a strong relationship between egg yolk color and egg quality. A dark yolk color implies better egg quality; therefore, eggs with a darker yolk color are preferred by consumers (43, 44). The carotenoid content of the laying hens diet is the main factor affecting the yolk color. Several studies have shown that carotenoids found naturally in plant-based diets of laying hens are transferred to the yolk of the eggs laid. Different diet components can also influence the yolk's color, such as the lipid structure and the type and amount of carotenoids (45–47). The darker yolk color in this experiment is presumed to be due to the impact of MLE on the lipid metabolism of laying hens, promoting both the absorption of fat-soluble carotenoids and their deposition in the yolk.

The liver is an essential organ for lipid metabolism in poultry and an integral part of the *ab initio* synthesis of fatty acids, with nutrients entering the liver through the portal vein after absorption in the small intestine (48). The diet of laying hens contains only a small amount of cholesterol, and the cholesterol of body is mainly synthesized through the liver; two-thirds of the cholesterol is metabolized through eggs, and the rest is metabolized through fecal and bile acid metabolic pathways. In this experiment, TG and TC contents were significantly reduced in both the liver and egg yolk of the test groups, further indicating that MLE positively affects lipid metabolism in poultry. Most plant polysaccharides have hypolipidemic effects. The addition of 1–2 g/kg mannan-oligosaccharides to the diet significantly reduced serum TG and LDL contents in laying hens (49). The addition of 0–20 g/kg sumac and ginger to the diet significantly reduced TC contents in egg yolk and serum (50). To further validate these results, we measured the protein expression of relevant lipid metabolism genes in the liver in response to available experimental data.

Peroxisome proliferators-activated receptors (PPARs) are ligand-activated receptors in the nuclear hormone receptor superfamily and are present as three isoforms (51). PPARα is the main transcription factor that regulates mitochondrial fatty acid β-oxidation genes and is negatively correlated with IMF content (52). PPARγ promotes liver energy storage and adipocyte differentiation and is potentially regulated by SREBP-1C to regulate lipid synthesis (53, 54). SREBP-1C preferentially regulates the biosynthesis of fatty acids, phospholipids, and triglycerides and can activate the fatty acid synthase gene (FASN) (55). FASN is a rate-limiting enzyme for fat regeneration capacity and is involved in fat deposition and phospholipid synthesis in animals; its elevated expression level leads to a significant increase in triglycerides *in vivo* (56). Silent information regulator 1 (SIRT1) is an NAD^+^-dependent deacetylase involved in regulating lipid metabolic processes, acts as a negative regulator of TG synthesis, and is capable of stimulating fatty acid oxidation (57). Our experimental results showed that MLE might affected liver lipid metabolism in laying hens by influencing the SIRT/PPAR signaling pathway. Additionally, it reduces the expression of its target gene-FASN by inhibiting the expression of the transcription factor SREBP-1C, thereby reducing lipid synthesis. It has been shown that 0.8 g/kg·d MLP inhibited adipocyte differentiation and triglyceride synthesis by affecting the PPAR-γ-C/EBP-α signaling pathway in rats (58), and the addition of 5% MLP to the diet of fattening pigs resulted in a decrease in FAS and a significant increase in hormone-sensitive adiponectin and leptin receptors (59). Other plant extracts can also affect lipid metabolism in livestock by modulating the SIRT/PPAR pathway. Green tea extract can reduce abdominal fat accumulation in broiler chickens by downregulating PPARγ expression in abdominal adipose tissue (60). The addition of genistein to laying hen diets inhibits fatty acid synthesis and enhances β-oxidation in the liver by modulating the PPAR-LXRα-SREBP1c-ACC/FAS/FAT pathway (61).

## Conclusion

In conclusion, adding 0.8% MLE to the diet of laying hens could improve egg quality and antioxidant capacity, regulate lipid metabolism, reduce the probability of lipid metabolism-related diseases in the egg-laying period, and extending the egg-laying cycle, Obtain higher economic benefits when promoting the application in the future. The study will provide a theoretical reference for the application of MLE in egg production and promoting the healthy and sustainable development of the livestock and poultry industry under the background of antibiotic prohibition.

## Data availability statement

The datasets presented in this study can be found in online repositories. The names of the repository/repositories and accession number(s) can be found in the article/supplementary material.

## Ethics statement

The animal study was reviewed and approved by the Animal Use and Ethical Committee of Hebei Agricultural University (University Identification Number: HB/2019/03). Written informed consent was obtained from the owners for the participation of their animals in this study.

## Author contributions

BZ, DC, HC, and ZW: design and complete the experiment. BZ, DW, HC, and YC: statistics and contributions. HC and XS: provide experimental guidance. All authors contributed to the article and approved the submitted version.

## Funding

This study was supported by the China Agriculture Research System of MOF and MARA (CARS-40), the S&T Program of Hebei (20326609D) and the S&T Program of Hebei (22327506D).

## Conflict of interest

The authors declare that the research was conducted in the absence of any commercial or financial relationships that could be construed as a potential conflict of interest.

## Publisher's note

All claims expressed in this article are solely those of the authors and do not necessarily represent those of their affiliated organizations, or those of the publisher, the editors and the reviewers. Any product that may be evaluated in this article, or claim that may be made by its manufacturer, is not guaranteed or endorsed by the publisher.

## Abbreviations

PPARα: peroxisome proliferators-activated receptor-α
PPARγ: peroxisome proliferators-activated receptor-γ
SIRT1, Silent information regulator 1;FASN: fatty acid synthetase
SREBP-1c(SREBF1): sterol regulatory element-binding protein-1c
ALB: albumin
GLB: globulin
TG: triglyceride
TC: total cholesterol
VLDL-C: very-low-density lipoprotein
LDL-C: Low-Density Lipoprotein
HDL-C: high-density lipoprotein
ALT: alanine transaminase
AST: aspartate transaminase
CAT: catalase activity
T-AOC: total antioxidant capacity
SOD: superoxide dismutase
GSH-Px: glutathione peroxidase
MDA: malondialdehyde.
